# Public Health Service Discovers Cause and Cure of Pellagra

**Published:** 1915-12

**Authors:** 


**Public Health Service Discovers Cause and Cure of Pellagra. Pellagra Caused by Insufficient Proteid Diet** . Pellagra has been increasing alarmingly throughout the United States during the last eight years, and it is estimated that 75,000 cases of the disease will have occurred in the United States in 1915, and of this number at least 7,500 will have died before the end of the year. In many sections only tuberculosis and pneumonia exceed it as a cause of death.

The final epoch-making experiment of the Public Health Service was carried out at the farm of the Mississippi State Penitentiary about eight miles east of Jackson, Miss., and together with the previous work of the Service completes the chain in the prevention and cure of the disease. The work at the Mississippi Farm has been in charge of Surgeon Joseph Goldberger and Assistant Surgeon G. A. Wheeler of the United States Public Health Service. The Farm consists of 3,200 acres in the center of which is the convict camp. The final experiment was undertaken for the purpose of testing the possibility of producing pellagra in healthy human white adult males by a restricted, one-sided, mainly carbo-hydrate (cereal) diet. Of eleven convicts who volunteered for this experiment, six developed a typical dermatitis and mild nervous gastro-intestinal symptoms.

Experts, including Dr. E. H. Galloway, the Secretary of the Mississippi State Board of Health, Dr. Nolan Stewart, formerly Superintendent of the Mississippi State Hospital for the Insane at Jackson, Dr. Marcus Hause, Professor of Dermatology, Medical College of the University of Tennessee, Memphis, Tenn., and Dr. Martin R. Engman, Professor of Dermatology in the Washington Medical School, St. Louis, Mo., declare that the disease which was produced was true pellagra.

Prior to the commencement of these experiments no history could be found of the occurrence of pellagra on the penitentiary farm. On this farm are 75 or 80 convicts. Governor Earl Brewer offered to pardon twelve of the convicts who would volunteer for the experiment. They were assured that they would receive proper care throughout the experiment, and treatment should it be necessary. The diet given was bountiful and more than sufficient to sustain life. It differed from that given the other convicts merely in the absence of meats, milk, eggs, beans, peas, and similar proteid foods. In every other particular the convicts selected for the experiment were treated exactly as were the remaining convicts. They had the same routine work and discipline, the same periods of recreation and the same water to drink. Their quarters were better than those of the other convicts. The diet given them consisted of biscuits, fried mush, grits and brown gravy, syrup, corn bread, cabbage, sweet potatoes, rice, collards and coffee with sugar. All components of the dietary were of the best quality and were properly cooked. As a preliminary, and to determine if the convicts were afflicted with any other disease, they were kept under observation from February 4th to April 9th, two and a half months, on which date the one-sided diet was begun.

Although the occurrence of nervous symptoms and gastro-intestinal disturbances was noted early, it was not until September 12th, or about five months after the beginning of the restricted diet, that the skin symptoms so characteristic of pellagra began to develop. These symptoms are considered as typical, every precaution being taken to make sure that they were not caused by any other disease. The convicts upon whom the experiment was being made, as well as twenty other convicts who were selected as controls, were kept under continuous medical surveillance. No cases of pellagra developed in camp excepting among those men who were on the restricted diet. The experimenters have therefore drawn the conclusion that pellagra has been caused in at least six of the eleven volunteers as a result of the one sided diet on which they subsisted.

On the basis of this discovery, the States of Mississippi, Louisiana and Florida have laid their propaganda through their respective boards of health for the eradication of the disease.

With reference to the discovery of the cause of Pellagra, we quote from a series of articles on European Travel, by Dr. F. H. Hamilton (Buffalo Medical Journal, Dec. 1845): "The causes of this scourge of Lombardy are, I am convinced and so also I was assured by Dr. Capelli, to be found not only in the low damp soil of the adjacent country and the exposure to a burning sun, but chiefly in the peculiarly unwholesome diet of the peasantry, whose food, exclusively vegetable, is chiefly composed of Indian flour made into a coarse and always acid bread......which never failed to put my stomach into a fermentation; or Indian flour and chestnut flour stirred into a porridge called 'polenta' and to which the rye porridge of Scotland is not unlike........ The treatment at this hospital (Ospedale Maggiore, Milan) consists mainly in a plain, healthy, nutritious diet and baths." The present editor has, from the beginning of the agitation of the subject of Pellagra in the American medical literature, contended that it was not a specific disease, not even in the sense of being especially connected with maize or a fungus or bacterium developing in it, but that it was a symptom complex of nutritional disturbance. We note with pleasure the confirmation and more accurate demonstration of views long held by the profession, but the word Discovery does not seem to us appropriate.

